# Efficacy and safety of omalizumab treatment to facilitate successful oral desensitisation in high-risk milk-allergic patients

**DOI:** 10.1186/2045-7022-5-S3-P40

**Published:** 2015-03-30

**Authors:** Rita Aguiar, Ana Celia Costa, Manuel Pereira-Barbosa

**Affiliations:** 1 Immunoallergology Department, Lisbon, Portugal

## Background

One of the most promising therapies in persistent and severe food allergy is oral immunotherapy (OIT), with immediate goal of desensitization and long-term goal of tolerance. However, a subgroup of patients may experience severe allergic reactions. These concerns might be addressed by combination of OIT with another promising therapy involving anti-IgE monoclonal antibodies (omalizumab), which can reduce allergic reactions associated with food administration.

## Case report

*Case1:* Female, 16 years, with severe IgE-mediated cow's milk allergy (CMA), allergic asthma, rhinitis and eczema. From 6 months, after the 1st contact with milk, she suffered multiple anaphylaxis after exposure to hidden cow's milk allergens, despite the restrictive eviction. She was submitted to milk OIT in day hospital, having been able to reach 40ml of pure cow milk, with mild adverse reactions that were controlled. After 1 month of daily milk intake, she developed persistent abdominal pain, difficulty breathing and a feeling of tightness neck that were worsening with angioedema of the face even with reducing the milk dose to 10ml and under medication with anti-H1, anti-H2 and montelukast and then self-suspending milk ingestion. She started omalizumab 225mg SC 2/2 weeks(w) and re-initiated OIT, with oral ingestion of increasing doses of CM until reaching the target of 120ml/day and now she eat some milk manufactured products(cheese, butter).

*Case2:* Male, 24 years, with severe IgE-mediated CMA, with symptoms since the 1st day in maternity hospital (difficulty breathing after taking the 1st milk aby bottle).Since then, he had several anaphylactic reactions with milk traces despite strict diet. He initiated milk OIT reaching 0.2 ml (dilution 1/10) for 3 weeks. However, he triggered an anaphylactic reaction controlled with adrenaline. Then, he started omalizumab 225mg SC 2/2 w and initiated OIT gradually until 20ml.

Without adverse reactions associated to omalizumab.

## Discussion

A successful cow's milk OIT was possible with the administration of omalizumab. It showed to be effective and safe, reducing the risk of severe reactions and improving the quality of life of pts and their family.

## Consent

Written informed consent was obtained from the patient for publication of this abstract and any accompanying images. A copy of the written consent is available for review by the Editor of this journal.

